# Quality Control of DAS VSP Data in Desert Environment Using Simulations and Matching Filters

**DOI:** 10.3390/s24041075

**Published:** 2024-02-07

**Authors:** Nour Alzamil, Vladimir Kazei, Huawei Zhou, Weichang Li

**Affiliations:** 1 Department of Earth and Atmospheric Sciences, University of Houston, 4800 Calhoun Rd., Houston, TX 77004, USA; hzhou3@central.uh.edu; 2 EXPEC Advanced Research Center, Saudi Aramco, Dhahran 31311, Saudi Arabia; 3 Aramco Research Center—Houston, Aramco Americas, 16300 Park Row, Houston, TX 77084, USA; vladimir.kazei@aramcoamericas.com (V.K.); weichang.li@aramcoamericas.com (W.L.)

**Keywords:** distributed acoustic sensing, data quality control, vertical seismic profiling, depth calibration, well logs

## Abstract

The unconsolidated near surface and large, daily temperature variations in the desert environment degrade the vertical seismic profiling (VSP) data, posing the need for rigorous quality control. Distributed acoustic sensing (DAS) VSP data are often benchmarked using geophone surveys as a gold standard. This study showcases a new simulation-based way to assess the quality of DAS VSP acquired in the desert without geophone data. The depth uncertainty of the DAS channels in the wellbore is assessed by calibrating against formation depth based on the concept of conservation of the energy flux. Using the 1D velocity model derived from checkshot data, we simulate both DAS and geophone VSP data via an elastic pseudo-spectral finite difference method, and estimate the source and receiver signatures using matching filters. These field geophone data show high amplitude variations between channels that cannot be replicated in the simulation. In contrast, the DAS simulation shows a high visual similarity with the field DAS first arrival waveforms. The simulated source and receiver signatures are visually indistinguishable from the field DAS data in this study. Since under perfect conditions, the receiver signatures should be invariant with depth, we propose a new DAS data quality control metric based on local variations of the receiver signatures which does not require geophone measurements.

## 1. Introduction

In 1880, Alexander Graham Bell transmitted the first message converting acoustic vibrations into light in his photophone. Yet, it was not until the 1960s, when experiments by Charles Kuen Kao and others with lasers and glass fibers gave rise to the modern broadband communication and massive deployment for fiber-glass optic cables [1]. Over the past two decades, a new technology based on the optical time domain reflectometer has arisen based on one of Bell’s ideas of converting vibrations into light. This new technique used in borehole geophysics, often for vertical seismic profiling (VSP) is called distributed acoustic sensing (DAS) [2,3]. This technique uses optical fibers, which are made of glass with a very thin diameter that can reach 9 μm, to act as sensors recording seismic waves propagating in the subsurface. The number of wells with fiber optic (FO) cables installed has increased noticeably due to their advantages for sensing temperature, pressure, and fluid flow. In particular, the FO widens the frequency bandwidth over that of conventional geophones by at least 17 octaves [4]. With a minor operational cost and low power consumption, FO sensing can be used on-demand to acquire single or multiple sets of VSPs covering the full depth range of the well without well intervention [5,6,7]. Since dense sampling by borehole receivers is beneficial for large-scale 3D VSP imaging in the Middle East [8], it is highly desirable to replace geophones by DAS to achieve dense channel VSP imaging at affordable cost [9]. However, concerns on the quality of DAS data have held back the deployment of DAS in VSP surveys and demanded good solutions on quality control (QC). Comparing with the geophone data, the DAS VSP data often have issues in noise level, channel depth uncertainty, and measurement amplitude reliability.

The noise characteristics of DAS data often differ from that of geophone data, as shown in QC studies (e.g., [10,11,12,13]) and signal enhancement works (e.g., [14,15,16]). A major noise contributor is from the field conditions of DAS VSP surveys. In particular, desert environment features large daily temperature variations and extremely low velocities and high heterogeneity in the near surface. In fact, VSP is often chosen for seismic imaging in the desert because it is less susceptible to near surface velocity anomalies [8,17]. Still, only recently a successful multi-well–single interrogator unit (IU) DAS VSP acquisition was demonstrated by Aldawood et al. [18] in a desert environment. 

To assure data quality, depth uncertainty, and DAS amplitude, the reliability must be properly addressed when deploying DAS. Especially, any poor coupling of the sensors in the wellbore should be revealed by QC procedures. One of the challenges to using DAS data is calibrating the depth of DAS channels in the wellbore to the formation depth. Overstuffing a cable with optical fiber, cable relaxation between clamps, excessive cable length at the well head, fiber refractive index measurement imperfection, or inaccurate depth of the end of the fiber are leading factors contributing to depth calibration errors in DAS data [19,20,21]. Bakku [22], Ellmauthaler et al. [23], and Wu et al. [24] developed techniques to overcome the depth calibration challenge, mainly, by referencing the depth of DAS channel depth in the wellbore to certain features with known positioning such as plug depth, perforation shots, tubing clamps, or the end of the cable. However, these features can still be uncertain due to, for example, uneven fiber distribution between these reference features, subsurface compression, or fiber breakage. Olofsson and Martinez [21] suggested another way to resolve the relative depth calibration error with the assumption of knowing the velocity field. DAS channels are first migrated individually after interchanging the source location as receivers and DAS channels as shots. Then, the migrated volumes are cross-correlated to measure the relative depth differences between these DAS channels. Mateeva and Zwartjes [20] developed a simpler method that relates DAS measurement amplitudes to a well log upscaled acoustic quantity in order to calibrate DAS channel scaler depth to the geology. Kazei and Osypov [25] and Pevzner et al. [26] extended Mateeva’s method to property estimation techniques, which used DAS depth calibration here.

In this paper, we focus on developing practical solutions for DAS data QC and signal enhancement, using the same dataset of that by Aldawood et al. [18]. In the following, we show the acquisition of the DAS data and our analysis in DAS depth calibration and estimation on source signature and instrument response. Based on numerical modeling using well logs and checkshot velocities, our simulated DAS data achieve a high similarity with the field DAS data. This suggests a new QC tool for DAS VSP data in desert environment via simulations using well logs, without geophone data.

## 2. Field DAS Data Acquisition and Previous Findings

**Figure 1:**
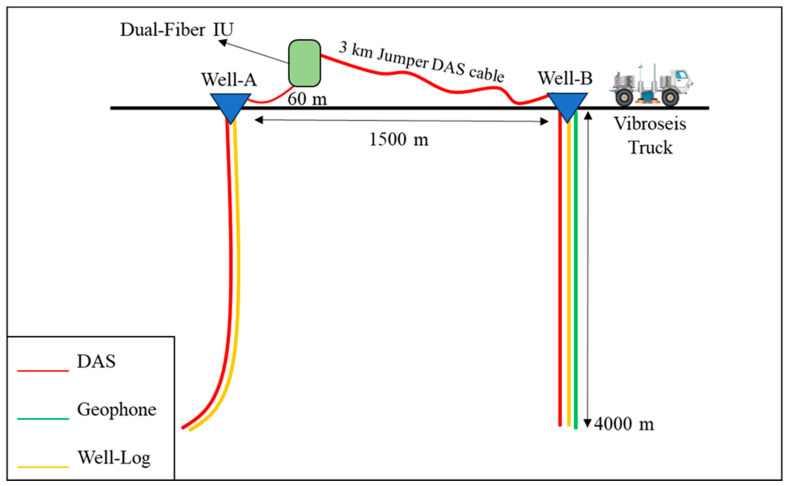
A sketch showing the dual-fiber IU connecting through a jumper DAS cable with two SM fiber optic cables that are clamped on tubing in two wells. Well log and DAS data are acquired from well-A while well log, DAS and geophone are obtained from well-B.

The field data were acquired in a desert environment with two adjacent non-flowing wells extending to about 4 km depth, following an acquisition geometry shown in Figure 1. The wells are separated by 1.5 km and equipped with pre-installed FO cables. A vibratory source was used while acquiring VSP data with two sensing systems which are distributed acoustic sensing and the conventional system using geophones. One of the wells (well-A) has fiber optic cable installed inside the borehole besides obtaining sonic log data, while the other well (well-B) has three different installations that provide DAS, geophone, and well log data. Well-A is highly deviated especially at the deeper section, in contrast to the mostly vertical well-B where the maximum inclination is 2.2 degrees.

The DAS system relies on fiber optic cables that are clamped on tubing on both wells, which are connected through a jumper DAS cable on the surface to a dual-fiber interrogator box. The geophone system is an array of vertical particle velocity sensors deployed in well-B, which provides well log data. Since our analysis focuses on well-B, we refer to it as “the well” in further text. A summary of the geophone and DAS acquisition and recording parameters are given in Table 1, showing the source, geophone, and DAS recording parameters in the acquisition. Illustration of the acquisition geometry and the data type of each well are shown in Figure 1, where DAS fiber optic cable is in red, geophone is in green, and the well log is in yellow.

According to Aldawood et al. [18]. this survey was initially designed to acquire DAS dual-well walk-away VSP data simultaneously, in order to assess the quality of the data, seismic reflectivity images, and velocity model building based on DAS, geophone, and well logs. Their main findings include the following: 

Well-B showed a 5 dB reduction in the signal-to-noise ratio (SNR) in comparison to well-A due to the long jumper DAS cable that connects well-B fiber to the interrogator box.

DAS corridor stack from both wells and geophone corridor stack showed good agreement with the surface seismic.

DAS velocity profile at well-B can be used to reconstruct a high resolution P-wave velocity (V_p_) model that agrees with the sonic log.

The jumper cable connecting the interrogator unit with well-B is 3 km long which would typically lead to 1–2 dB SNR reduction [10]. Aldawood et al. [18] reported 5 dB reduction due to fiber imperfections near the wellhead confirmed by time-domain reflectometry tests. Nevertheless, we still find DAS data are more reliable than geophone data for the same well. The gauge length in this experiment was set to 24 m to increase the SNR and to capture signals at deeper sections. Two vibroseis trucks were used simultaneously as a source, performing 16 sweeps to acquire the DAS data with a dense sampling of 6.4 m receiver spacing to enhance first break picking and other signal processing steps. 

**Figure 2:**
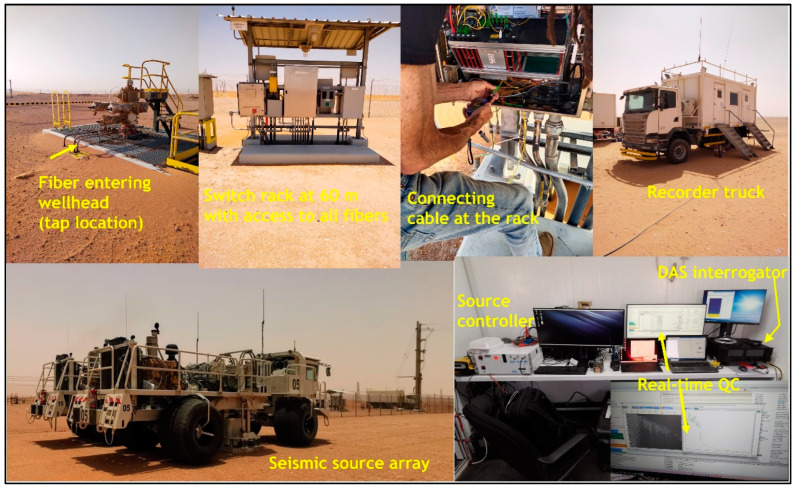
Photos of the dual-well DAS VSP acquisition survey show the tap location at the wellhead, switch rack at 60 m from well-A with a fiber connection, recorder truck, seismic source array for the DAS walkaway VSP, source controller, and DAS interrogator with other equipment in the recording room [18].

The photos of the dual-well DAS VSP acquisition survey in Figure 2 show the tap location at the wellhead, switch rack at 60 m from well-A with a fiber connection, recorder truck, seismic source array for the DAS walkaway VSP, source controller, as well as DAS interrogator and other equipment in the recording room. The VSP data acquired were correlated and stacked to enhance the SNR.

For the geophone dataset, the GEOCHAIN-ASR (advanced seismic receivers) system was used which is a system that is designed for open or cased holes with all locking arms opening simultaneously to reduce survey time. Single-component (vertical) geophones were used to acquire zero offset VSP data in well-B. This dataset was acquired prior to the DAS experiment with one vibroseis truck. The geophone array consists of a 4-channel string with a channel spacing of 15 m. The string was lowered into the borehole to acquire 248 channels. Unfortunately, the geophone data quality is poor overall as two out of the four receivers suffered from amplitude discrepancies.

## 3. DAS Depth Calibration

Mateeva and Zwartjes [20] developed a simpler method that relates DAS measurement amplitudes to a well log upscaled acoustic quantity to calibrate DAS channel scaler depth to the geology. The fact that the strain measurement by DAS has a linear relationship with an acoustic quantity from logs establishes a basis for quantitatively calibrating DAS VSP measurements to formation depth. Specifically, we can relate the amplitude of DAS data to a well log upscaled acoustic quantity that is more sensitive to velocity variation. For DAS depth calibration in this paper, we follow the inversion approach by Pevzner et al. [26] who used synthetic and field DAS data to demonstrate a linear relationship between DAS measurement (strain) amplitude and an acoustic quantity ${\left(\rho *c^{3}\right)}^{-0.5}$ composed of medium density ρ and velocity c. This relationship is based on the energy flux conservation theory and neglects the transmission loss of the acoustic impedance variations within each wavelength. Therefore, we can use the DAS amplitude variation to estimate the formation acoustic properties.

Our objective here is to align the channel depth of DAS and geophone measurements with the formation depth based on well logs, following a modified methodology of [20]. In the following, we list the steps of an amplitude-based DAS depth calibration procedure, followed by an explanation.

- Picking the first break arrival times
- Flattening DAS and geophone first arrivals with the first break picks (FBPs)
- Specifying 50 ms window around FBPs for DAS and geophone to compute RMS amplitudes
- Calculating V_p_ proxies from DAS and geophone using RMS values and picked traveltimes; computing windowed cross-correlation of P-wave velocity from DAS with log and geophone with log for different depth shifts
- Extracting the depth shift with the highest correlation coefficient
- Applying that depth shift to DAS channels to maximize the correlation.

**Figure 3:**
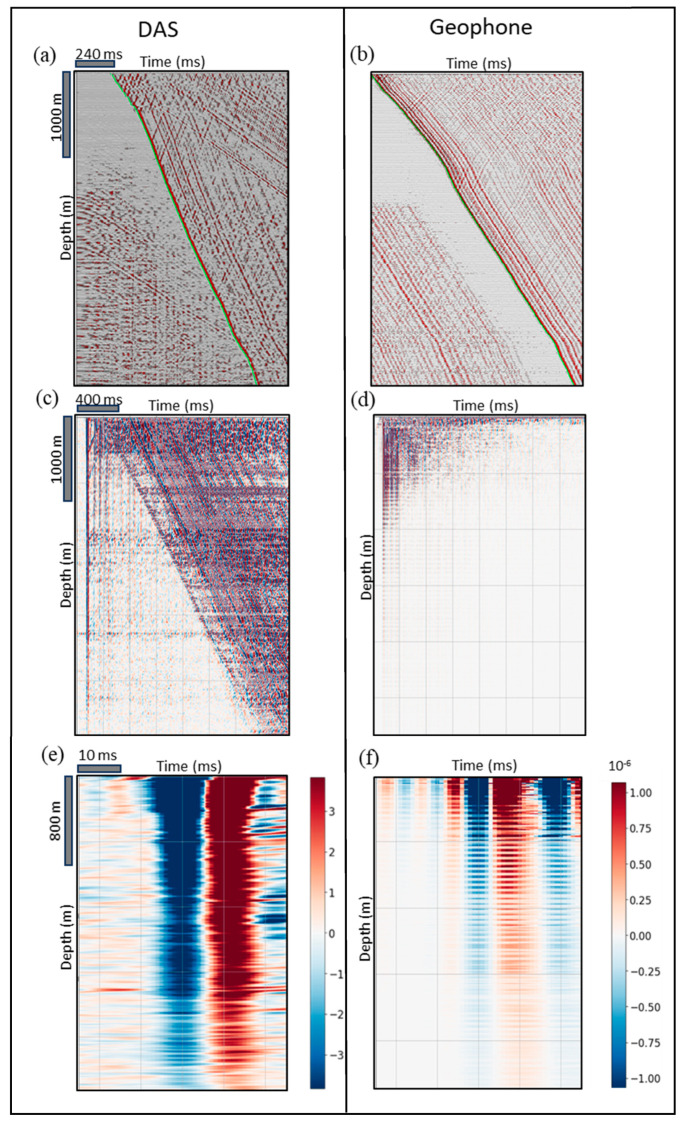
Zero offset shot records of (**a**) DAS and (**b**) geophone data where green curves denote the first break picks. The shot records after flattening the first arrivals for (**c**) DAS and (**d**) geophone. A time window of 50 ms around the first break picks for (**e**) DAS and (**f**) geophone data.

The first three steps above are rather routine for VSP data analysis, and the results of these steps are combined in Figure 3; started by picking the first break arrivals of the zero offset DAS and geophone shot records at Figure 3a,b. Note that, in Figure 3b, some coherent noise is shown on the upper right corner. Due to automatic gain control (AGC) effect, which is one of the most common gain recovery methods in seismic processing, this coherent noise before the first break arrivals is amplified and appears to look like signals. However, when computing this noise, it is correlation duplication. A similar effect also appears in the upper right corner of Figure 3a as horizontal lines before the first break arrivals, and it is called instrument noise. Typically, a noise shakes the IU and the box puts that noise across all DAS data. In Figure 3c,d, the first break arrivals data were flattened by shifting traces with picked travel times. After that, a time window of 50 ms around FBPs was selected to compute the root-mean-square (RMS) amplitudes for DAS measurement (strain) and the amplitude of geophone measurement (particle velocity). Figure 3e,f displays the windowed flattened DAS and geophone sections. It is obvious from Figure 3f that the geophone absolute amplitudes have high variations due to poor coupling of two out of four actual devices used for acquisition making them unusable.

**Figure 4:**
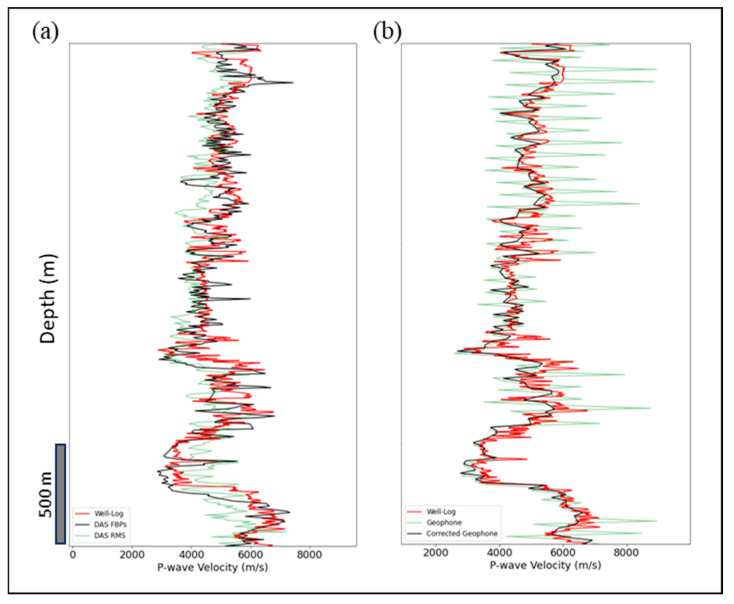
Comparison of P-wave velocities with depth between DAS, geophone and well log data. (**a**) Black curve represents DAS velocity estimated from the FBPs, green is velocity estimated from DAS RMS amplitudes, and red is for upscaled well log velocity. (**b**) Green curve represents geophone velocity estimated from FBPs, black is geophone corrected velocity, and red is for upscaled well log velocity.

**Figure 5:**
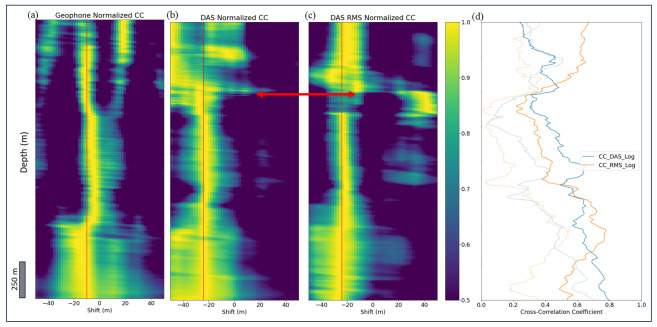
Three panels of normalized cross-correlation coefficients (in 500 m depth window) as functions of depth and depth shift between log-based V_p_ and (**a**) time-based geophone V_p_, (**b**) time-based DAS V_p_, and (**c**) amplitude-based DAS V_p_, respectively. Note in (**b**) that −24 m depth shift for DAS data (shifting channels up) delivers the best match to log-based data; (**d**) cross-correlation curves (unnormalized) between log-based V_p_ and time-based DAS V_p_ (CC_DAS_Log); and amplitude-based DAS V_p_ proxy (CC_RMS Log) before (transparent curves) and after (solid curves) applying the −24 m depth shift.

Following Pevzner et al. [26], we estimated V_p_ from DAS RMS amplitudes. Figure 4a shows the V_p_ proxy against the upscaled well log V_p_ and DAS interval V_p_. Since the amplitudes of geophone data have unrealistic variations (Figure 3f), we only extracted the interval velocity from the FBPs of the geophone data and compared them with the DAS and upscaled log V_p_ in Figure 4. The green curve in Figure 4b reflects the issue with two out of four geophones in the acquisition, noted by the acquisition team. This results in a quasi-periodic variation with a period of about 60 m, corresponding to 4 channels shift. Non-regularized geophone picks (green curve in Figure 4b) result in geologically non-feasible P-wave velocities that can be corrected in processing [18], (black curve in Figure 5b). The velocities from DAS RMS amplitudes are less biased to the interpreter’s FBPs than the DAS interval velocity. Figure 4a shows that both velocities estimated from DAS RMS amplitudes and DAS FBPs match the upscaled well log. In Figure 4a, the DAS V_p_ from FBPs (black curve) shows a deeper shift compared to the well log (red curve), leading to our depth calibration shown in Figure 5. In Figure 4b, we notice that the well log (red curve) shows higher velocity on average than the corrected geophone velocity (black curve). This can be explained by the well-known typical increase in average P-wave velocity at higher frequencies confirmed by simulations [27] and case studies [28,29].

To quantify the quality of different V_p_ profiles from DAS and geophone data, we compute windowed cross-correlations treating well log V_p_ as ground truth. The highest cross-correlation corresponds to the optimal local depth shift to align VSP measurements and the well log. Figure 5 shows cross-correlation coefficients normalized to have a maximum value of 1 at each depth. The red line in Figure 5b,c represents the −24 m bulk shift that we applied to DAS data to maximize the correlation coefficient along most of the well depths. However, we observe that the shallow depth has a wider range of shift values. Figure 5d illustrates a comparison for the cross-correlation between DAS and the well log before and after the shift. There is also a large difference in depth shift values, around where the red arrow points, in Figure 5b,c which corresponds to low values of unnormalized cross-correlation coefficient in Figure 5d and likely indicates problems with coupling. For the geophone, as shown in Figure 5a, a bulk shift of −10 m gives the highest normalized correlation.

In this case, velocities estimated from either the DAS data or the DAS RMS amplitudes show a bulk depth shift of −24 m, or the DAS channel depth is deeper than the well log depths by 24 m, Interestingly, the bulk depth shift can be more reliably derived using the correlation with amplitude-based DAS velocity (Figure 5c) than that with time-based DAS velocity. In contrast, the bulk depth shift of geophones is −10 m. As shown in the bottom part of Figure 5a, there is not much variation in geophone shift values, whereas additional maxima at the top part occur, which might be caused by cycle skipping when matching P-wave velocities from log and geophones.

## 4. Source Signature and DAS Instrument Response Estimation

Many researchers [4,30,31,32] have derived empirical DAS instrument response R(t) that transfers true ground strain to observed DAS waveforms. Their derivations are based on comparisons between geophone and DAS data assuming a known and consistent geophone instrument response. In our case, the geophone data have inconsistent amplitude variations, hence cannot be used as a reference. Therefore, we propose to use synthetic simulations as a reference to expand the methodology of previous publications to the case where geophone data are unavailable or of poor quality. We represent the DAS data using the following convolutional model:

(1) $$U_{DAS}({t,\;x}_{r},\;x_{s})=R\left(t,x_{r}\right)*G\left(t,x_{r},x_{s}\right)*S(t,\;x_{s})$$

where $U_{DAS}$ is the recorded DAS data, R is the DAS instrument response, G is the DAS synthetics simulated with a Dirac-delta source (numerical Green’s function), and S is the source signature.

To analyze a newly introduced DAS instrument response, we first simulate synthetic elastic data and compare them with field DAS data. We then estimate the source signature using deconvolution. Next, we estimate the DAS instrument response connecting the synthetic and field traces at different depth levels using additional deconvolution operations. Finally, by applying the receiver’s response of a field shot gather (chosen at 50 m offset in this case) over a synthetic DAS shot gather that is simulated using a different offset (250 m) in combination with source estimation, we achieve an improved matching with the field record of the 250 m offset shot.

### 4.1. Simulation of DAS Data

Egorov et al. [33] developed a 2D pseudo-spectral seismic modeling code that simulates DAS seismic shot gathers using an elastic wave equation. In this study, DAS records of two shots were generated with a time sample interval of 5 ms and a grid size of 12.5 m. Sources were positioned at 50 m and 250 m offsets away from the wellhead. Acquisition with the FO cable along the whole borehole length with 6.4 m channel spacing and 24 m gauge length was simulated. These parameters replicate actual acquisition parameters described in Section 2. Data Type and DAS acquisition geometry. Dirac-delta function is used as a source signature to simulate Green’s function. Table 2 shows the modeling parameters including the source and receiver geometries.

**Figure 6:**
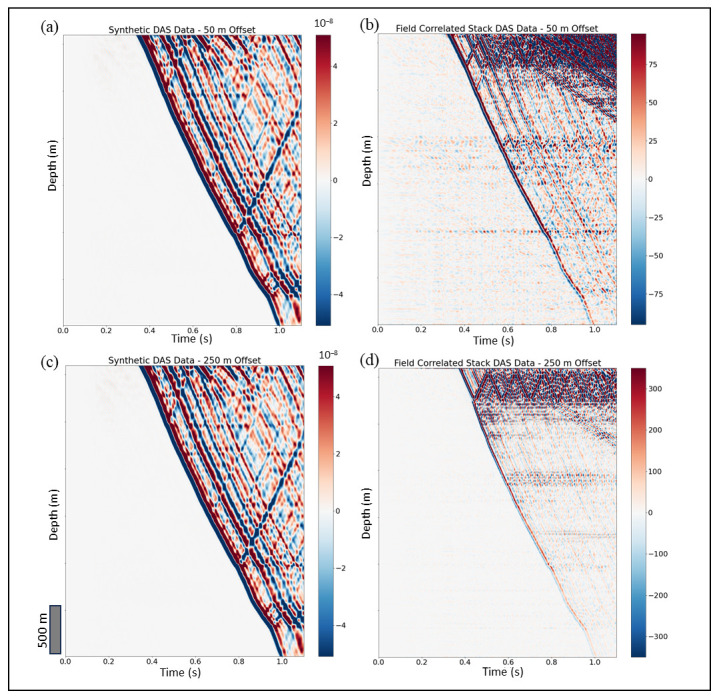
Synthetic of two DAS shot gathers of (**a**) 50 m and (**c**) 250 m offset, and field data of two DAS shot gathers of (**b**) 50 m and (**d**) 250 m offset.

The P-wave velocity generated using checkshot data for well-B (Figure 4b) is used as a key input parameter for DAS modeling. The S-wave velocity is estimated using constant V_p_/V_s_ ratio equal to 1.75. Gardner’s equation has been used to calculate the bulk density using V_p_. The source wavelet used for generating DAS synthetic data is a Dirac-delta wavelet. The synthetic DAS gathers of the two shots of 50 m and 250 m offset are illustrated in Figure 6a and Figure 6c, respectively. The output of the simulation is denoted as G. Convolution and deconvolution are the tools routinely used to match synthetic data and field observations. Using these tools, we show how to estimate source signature (S) and DAS instrument response (R) in the next two subsections.

### 4.2. Estimation of Source Signature

The source signature in a desert environment has typically poor repeatability [34], hence, it must be estimated for every vibrating point. Figure 6b,d shows two gathers of field DAS data for 50 m and 250 m offsets corrected for geometrical spreading, respectively. Also, note that the 1D model used for simulations is expected to work better for shorter offsets as 3D heterogeneity has less effects on vertically propagating waves. While there is resemblance in shape of the first arrival time curves of the corresponding synthetic gathers in Figure 6a (compared to 6b) and 6c (compared to 6d), the synthetic and recorded in-field wavefields cannot be compared without source matching.

**Figure 7:**
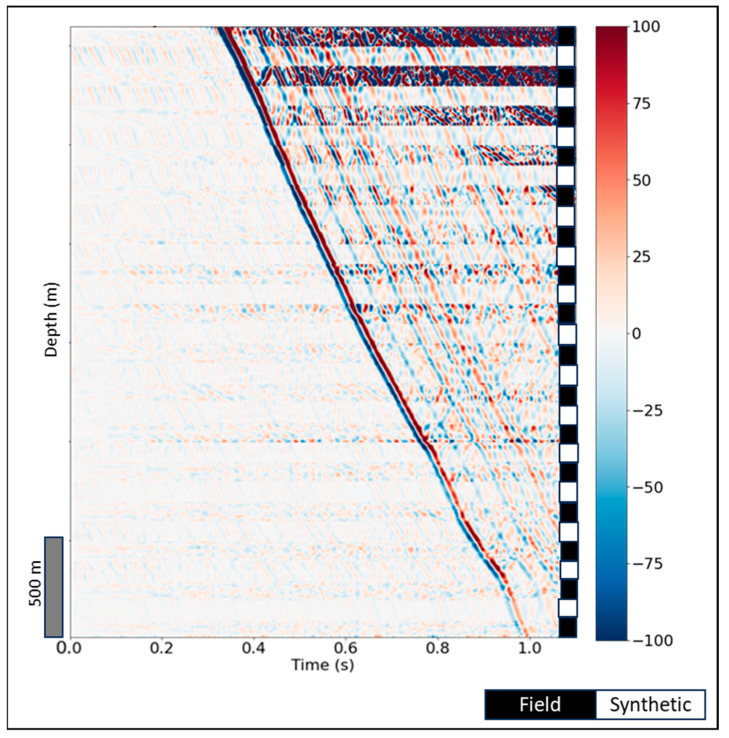
An interleaved DAS shot gather at 50 m offset for estimating the source signature using matching filter. The right stripe denotes field (black) and synthetic (white) data traces, respectively.

To compare field and synthetic DAS data directly, the source signature of synthetic DAS wavefield needs to be adjusted (Figure 7). We adjust the source by averaging convolutional matching filters (MF) constructed for trace-to-trace matching of 50 m synthetic and field DAS shot gathers.

**Figure 8:**
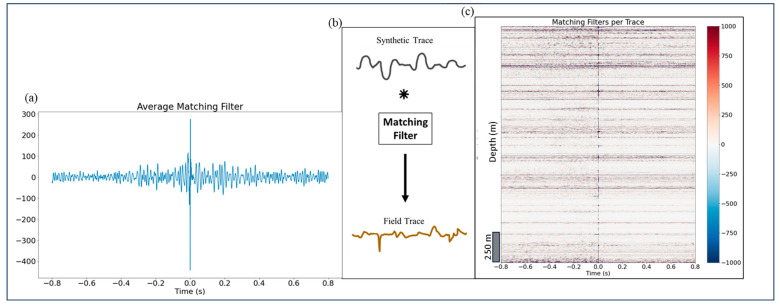
(**a**) DAS source signature estimated after using the matching filter, (**b**) scheme for mimicking the DAS field trace by convolving the synthetic trace with a matching filter, and (**c**) matching synthetic and field DAS data.

Figure 8a shows the DAS estimated source signature from using matching filter. The source signature was used as the S proxy in Equation (1) to generate synthetic traces in Figure 7. The scheme in Figure 8b illustrates how a matching filter connects synthetic trace with a field data trace via convolution. Finally, Figure 8c shows an example of matching filter which was computed by deconvolving field data traces with synthetic traces at the same depth.

**Figure 9:**
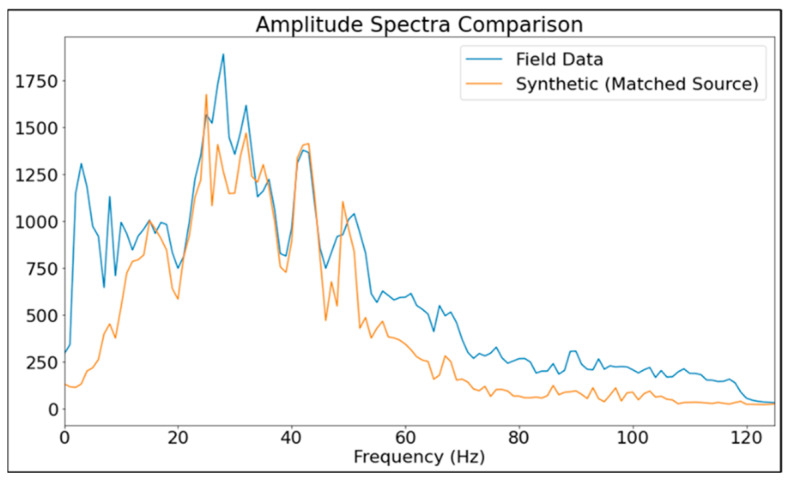
Amplitude spectra comparison between the synthetic (using matched source) and field DAS data.

To compare field and synthetic DAS data after using the matching filter to estimate the source signature, an amplitude spectra comparison between the synthetic (using matched source) and field DAS data is shown in Figure 9. It shows that the field data spectrum is relatively well replicated by synthetic data after source matching.

### 4.3. Estimation of Receiver Signature

**Figure 10:**
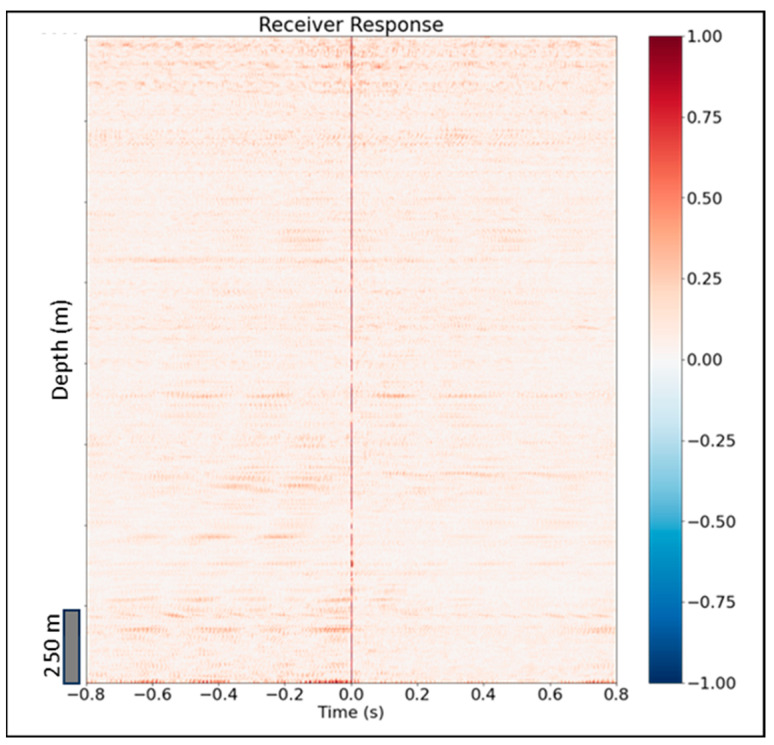
A 50 m offset shot receiver response shows almost no difference between field and synthetic DAS data.

Following Equation (1), to represent the DAS field, we need to estimate the instrument response R. We estimate R by deconvolving the DAS field wavefield with the synthetic DAS wavefield, trace by trace for a 50 m offset shot. Figure 10 illustrates our estimates of receiver instrument response for all channels. The ideal instrument response would be a delta function centered at time 0 indicating that the field data are perfectly replicated by the simulation, yet we observe variations in some channels. These discrepancies can be caused by local coupling issues with the fiber as well as inaccuracies in the elastic parameter model utilized for simulations.

**Figure 11:**
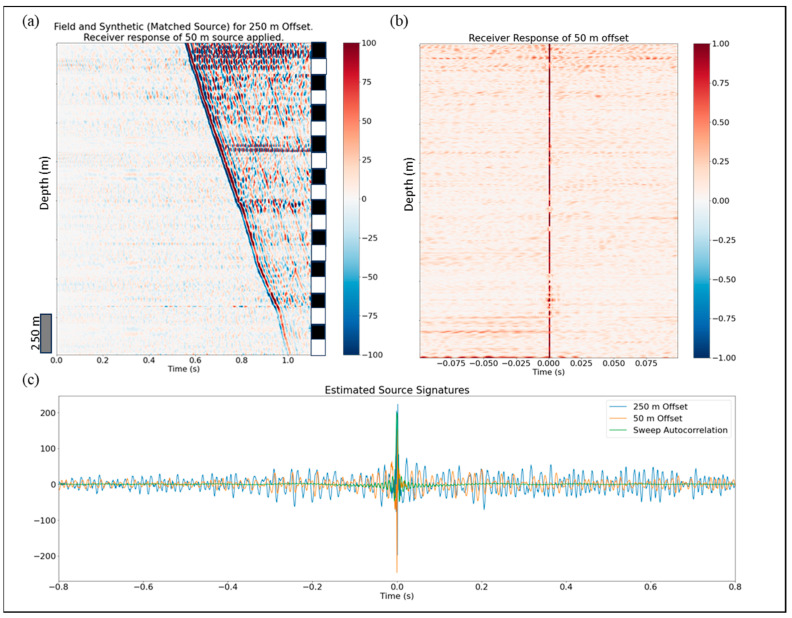
DAS data QC panel: (**a**) Field and synthetic data interleave after source and receiver signatures applied where the right stripe denotes field (black) and synthetic (white) data traces, respectively; (**b**) receiver response for 50 m offset; and (**c**) source signature comparison for 50 m and 250 m offsets.

Figure 11 shows the final QC panel that can be useful for DAS data quality assessment in the field. An interleave is shown in Figure 11a comparing the field and synthetic (matched source S and receiver response R applied) DAS data for a 250 m offset. Note that the receiver response was estimated using field data and synthetic data for a 50 m offset. One can barely distinguish the synthetic traces from the field in Figure 11a. Figure 11b shows a zoomed-in version of receiver response (Figure 10) where one can spot the amplitude variations with depth. In current implementation, the deconvolution is deterministic, and variations are only observed in the receiver function depending on spectrum thresholding that practically controls the variance. In future work this can be further studied by expanding the dataset and using more sophisticated deconvolution methods posing it as an optimization problem. Finally, Figure 11c demonstrates a comparison of source signatures estimated for both shot locations compared to an auto-correlation of the linear sweep of 2–120 Hz that was used by the vibroseis as input source signature in this field experiment. The sweep parameters were dictated by practicality. Higher frequencies quickly attenuate with depth due to scattering and intrinsic attenuation. Lower frequencies require very long total sweep durations to make the signal-to-noise ratio high enough for processing while each sweep already takes more than 20 s to record. While the timing of the peaks on the source signatures matches almost perfectly, a significant variation in the signal shape is observed, which is well supported by previous studies of [34] where they proved for the desert environment that running the source at the same location at different times, gives large variations in the source signature measured by a nearby geophone. The geophone data in this study were acquired with a source that is located in a different position and at a different time. This QC tool can be used in practice by looking at the receiver response, the channels that deviated from the mean above a certain threshold could be discarded from processing.

As a potential quantitative QC tool that can measure DAS VSP data, we add the peak signal-to-noise ratio for the receiver signatures. The peak of the signal is 1 assuming the noise is stationary around the peak, so the power is averaged in a small-time window. Averaging on time and receivers is denoted as $avg_{t}$ and $avg_{r}$, respectively:

(2) $$PSNR(x_{r})=10\log_{10}\frac{1}{avg_{t}(R\left(t,x_{r}\right)-avg_{r}{\left(R\left(t,x_{r}\right)\right)}^{2}}$$

**Figure 12:**
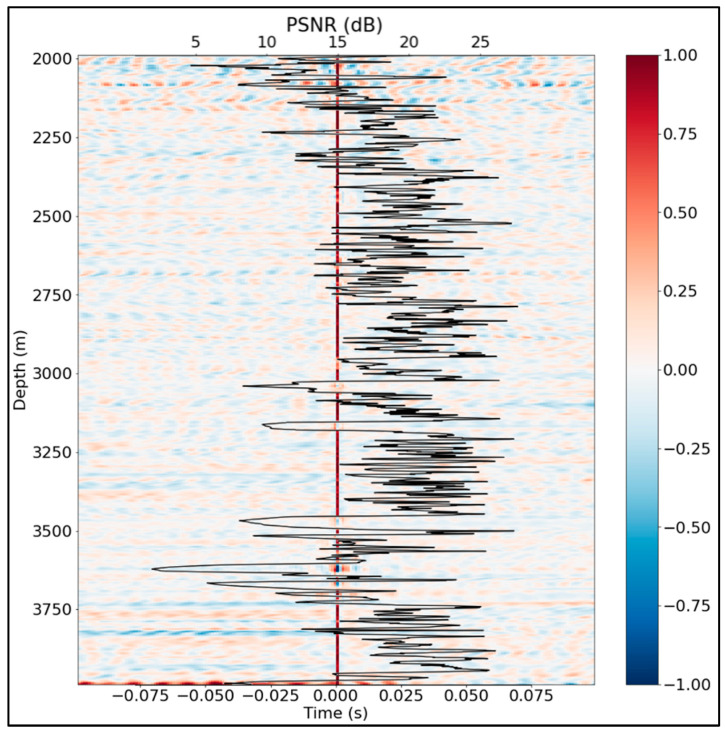
PSNR of receiver functions overlaying the receiver functions themselves.

Figure 12 shows that the noisy channels can be easily identified by simple thresholding of the new metric. Here the PSNR threshold of 15 dB appears to be a reasonable choice leading to poorly agreeing with synthetic simulation channel identification throughout the well.

PSNR just shows that a metric can be relatively easily defined to track the quality of the receiver functions and we do not expect that the exact definition expressed by Equation (2) will always lead to optimal results. Finetuning of such a metric can be the subject of future research. Notably such averaging of raw data is not possible and even if flattened, the data would suffer from variations in the wavelet with depth and reflections.

## 5. Discussion

Though geophone data are often considered as a ground truth for DAS data analysis, it is clearly not the case for our dataset as two out of four geophones used in this test had significantly different amplitudes and deemed the conversion between DAS and geophone data nonviable. In this case, depth and amplitude of the geophone data are inferior to the same attributes of the DAS data. For this dataset, the amplitude of geophones was not reproducible in the simulation. Without regularization, the first breaks from the geophone data led to wider spread in interval velocities. Both the geophone and DAS traces need depth shifts to match the logging depth. Contrary to DAS that can be adjusted with a single bulk shift over the measurement depth range, the FBP analysis of geophones suggests that the depth shift may vary with depth for optimal matching to logging data. The relative amplitudes of DAS data turned out to be more usable than the geophone amplitudes. Apart from the instrument coupling issues noted by the acquisition team for this particular survey, we can explain it by the difference in the nature of the sensors. Geophones are practically point receivers; hence, their amplitudes are heavily dependent on local properties of the subsurface. On the other hand, DAS is a distributed array of sensors which contributes to making the amplitudes of DAS measurements more robust.

Since the amplitudes of geophones were not reproducible in the simulation, we focused our analysis on DAS data. Instead of taking geophone data as the ground truth we used simulated DAS data to QC the field DAS data. Source signature estimation using matching filters led to a visual similarity in simulated and field DAS data for the first arrival waveforms at different offsets. This suggests that the 1D velocity model based on the checkshot data is an adequate representation of the subsurface for zero- and near-offset shot simulations in our case. Usage of 1D model for synthetic data simulations is an obvious limitation of the method, restricting it to only slowly, laterally varying areas. If large lateral variations are expected near the DAS-equipped borehole a 2D or even 3D model could potentially be built using VSP and potentially surface data. The 1D model itself can also potentially be further improved by incorporating the well log information into the model building process. However, a more advanced simulation model building process would also be more ambiguous. First, a more complex workflow would be needed to build a laterally inhomogeneous model. Second, part of the noise in the data could potentially be presented as lateral anomalies in the model. Finally, dispersion for P-wave velocity measured in logging and VSP experiments is a well-known but varying factor. Therefore, we acknowledge the simplicity of the 1D model used here as a limitation and leave potential improvements of the methodology for future research.

## 6. Conclusions

We observed that the desert environment with unconsolidated near surface can lead to non-replicable geophone amplitude variations. Hence, we designed a new QC workflow for DAS VSP data in the desert environment based on well logs, without the need for geophone data. DAS data simulation, using finite differences, and incorporating source and receiver signatures, leads to visually indistinguishable synthetic and field DAS data for the first arrival waveforms. Our work also demonstrates that significant amplitude variations in a few DAS channels can be replicated by including the instrument response or signature into the simulations.

## Figures and Tables

**Table 1:** Geophone and DAS acquisition and recording parameters during daytime in the summer.

Sensor	Seismic Source	Source Sweeps	Channel Spacing	Sampling Rate	Recording Length	Bandwidth	Gauge Length
Geophone	vibroseis	16	15 m	4 ms	4 s	2–120 Hz	-
DAS			6.4 m	1 ms			24 m

**Table 2:** The main input parameters for modeling two synthetic DAS gathers.

Input Parameters	Velocity Model (m/s)	V_p_
	Grid Size (m)	12.5
	Record Length (s)	4
	Time Sample Interval (s)	0.0005
Receiver Geometry	Rec. Orientation	Vertical
	Starting x of Rec. Array (m)	4000
	End x of Rec. Array (m)	4000
	Rec. Depth from Free Surface Start (m)	12.5
	Rec. Depth from Free Surface End (m)	4177
	Rec. Spacing Interval (m)	1
Source Geometry	Starting x of Source Array (m)	4050 and 4250
	End x of Source Array (m)	4050 and 4250
	Source Offset (m)	100
DAS Acquisition Parameters	Gauge Length (m)	24
Wavelet	Dirac-Delta	-

## Data Availability

Data associated with this research are confidential and cannot be released.
